# S100A12 facilitates osteoclast differentiation from human monocytes

**DOI:** 10.1371/journal.pone.0204140

**Published:** 2018-09-20

**Authors:** Miwa Nishida, Jun Saegusa, Shino Tanaka, Akio Morinobu

**Affiliations:** 1 Department of Rheumatology and Clinical Immunology, Kobe University Graduate School of Medicine, Chuo-ku, Kobe, Japan; 2 The Center for Rheumatic Diseases, Shinko Hospital, Chuo-ku, Kobe, Japan; 3 Clinical Laboratory, Kobe University Hospital, Chuo-ku, Kobe, Japan; Universite de Nantes, FRANCE

## Abstract

Osteoclasts play a critical role not only in bone homeostasis but also in inflammatory osteolysis, such as that occurring in inflammatory arthritis and systemic inflammation. In both inflammation conditions, inflammatory cytokines like Interleukin (IL)-1, IL-6 and tumor necrosis factor (TNF)-α induce RANKL expression in osteoblasts, but the roles of these cytokines in osteoclast activation remain unclear. S100A12, an S100 family member, is a low-molecular-weight calcium-binding protein. Although it has a pro-inflammatory role, its effects on osteoclast differentiation have been unclear. Here we examined the direct effects of S100A12 on human osteoclasts in vitro. S100A12 facilitated osteoclast formation in the presence of RANKL, as judged by the cells’ morphology and elevated expression of osteoclast-related molecules, including NFATc1, ACP5, CALCR, and ITGβ3. In addition, S100A12 administration markedly enhanced the osteoclasts’ bone resorption ability, consistent with their increased expression levels of CTSK and CA2. Blocking RAGE and TLR4 cancelled the effects of S100A12. Our results indicate that S100A12 is a potential therapeutic target for inflammatory osteolysis.

## Introduction

Osteoclasts originate from hematopoietic precursors of monocyte/macrophage lineage cells, which differentiate into multinucleated osteoclasts under the influence of macrophage colony-stimulating factor (M-CSF) and receptor activator of nuclear factor kappa-B ligand (RANKL). Osteoclasts play a critical role not only in bone homeostasis but also in inflammatory osteolysis [1, 2]. For example, the bone erosion in rheumatoid arthritis (RA) is a local osteolysis that is induced by the enhanced formation and activity of osteoclasts in affected joints. Inflammatory bone loss also includes the systemic bone loss that occurs in other inflammatory diseases such as inflammatory bowel disease, periodontitis, and chronic obstructive pulmonary disease [2]. In both inflammation conditions, inflammatory cytokines like Interleukin (IL)-1, IL-6, and tumor necrosis factor (TNF)-α, which are produced by activated macrophages in the inflamed tissue, play a critical role in osteoblast and osteoclast activation [1, 3].

The “alarmins” are endogenous substances that are released by dying cells or activated cells. They bind to certain pattern recognition receptors (PPRs) and induce tissue damage and/or inflammatory processes [4]. Representative alarmins include high-mobility-group box (HMGB) proteins, heat-shock proteins, and S100 family proteins [5]. There are 24 S100 protein family members, which are low-molecular-weight calcium-binding proteins that are predominantly expressed by neutrophils, monocytes, and activated macrophages. Their functions include the modulation of enzyme activity, calcium homeostasis, cell growth and mobility, cell differentiation, and cell survival. S100 proteins are reported to be involved in numerous human pathologies, including cancer, neurodegenerative diseases, Crohn’s disease, rheumatoid arthritis, Kawasaki disease, cystic fibrosis, and other inflammatory conditions [6–8].

Among the S100 proteins, S100A12 is constitutively expressed in neutrophils and macrophages. S100A12 is released from activated neutrophils, has proinflammatory effects on immune cells, and promotes inflammatory arthritis [9]. For example, Rouleau et al. revealed that the S100A12 expression is increased in the synovial fluids of patients with RA, gout, and psoriatic arthritis, but not osteoarthritis, a non-inflammatory disorder [10]. The serum S100A12 levels in RA patients are higher than those in healthy controls [11]. Moreover, Liao et al. detected elevated levels of S100A12 protein in both the synovial fluid and serum of patients with erosive forms of RA compared with those with non-erosive forms [12]. These findings suggest that S100A12 is significantly associated with both the inflammation and the bone erosion in RA.

Alarmins like HMGB1 and S100A8 have been shown to stimulate osteoclast formation and an activity mediating osteoclastic bone destruction in experimental arthritis [13]. However, the role of S100A12 in osteoclastogenesis has never been reported. Here we investigated the role of S100A12, a member of the S100 family, in osteoclast differentiation and activation.

## Materials and methods

### Reagents

Recombinant human M-CSF and recombinant human RANKL were purchased from R&D Systems (Minneapolis, MN). Recombinant human S100A8, S100A9, and S100A12 proteins were purchased from MBL (Aichi, Japan). The toll-like receptor 4 (TLR4) inhibitory peptide VIPER was from Novus Biologicals (Littleton, CO), and the anti-human receptor for advanced glycation end products (RAGE) antibody was from R&D Systems.

### Isolation and culture of CD14^+^ monocytes

The procedures were approved by the Ethics Committee of Kobe University Hospital (No. 160126), and oral informed consent was obtained from the healthy volunteers. Human peripheral blood mononuclear cells (PBMCs) were isolated from the blood of healthy donors, by centrifugation over Ficoll-Paque Plus (GE Healthcare, Uppsala, Sweden) at 1,500 rpm for 30 minutes. After centrifugation, PBMCs at the interface between the plasma and the Ficoll-Paque Plus were collected and washed with ice-cold phosphate-buffered saline, followed by centrifugation at 2,000 rpm for 5 minutes. CD14^+^ cells were isolated from PBMCs using positive selection with a MACS magnetic CD14 antibody (Miltenyi Biotec, Auburn, CA). The isolated CD14^+^ cells (1×10^5^/200 μl/well in a 96-well plate or 5×10^5^/1,000 μl/well in a 12-well plate) were incubated in α-minimal essential medium (α-MEM; Invitrogen, Tokyo, Japan) containing 10% fetal bovine serum, 100 units/ml penicillin, 100 μg/ml streptomycin (supplemented α-MEM), and 50 ng/ml M-CSF, with or without 100 ng/ml RANKL. S100A12 (1,000 ng/ml or as indicated elsewhere), S100A8 (1,000 ng/ml), and S100A9 (1,000 ng/ml) were added to cultures. Half of the medium was replaced every 3 days.

### Cell culture and inhibitor treatment

To induce osteoclast differentiation, CD14^+^ monocytes (5×10^5^ cells/12-well plate) were cultured in supplemented α-MEM containing M-CSF (50 ng/ml) and RANKL (100 ng/ml) for 7–16 days.

To elucidate the roles of TLR4 and RAGE in osteoclast differentiation and bone resorption, VIPER (30 μM) and/or an anti-human RAGE antibody (10 μg/ml) were used. In the bone resorption assay, VIPER (30 μM) and/or the anti-human RAGE antibody (10 μg/ml), and S100A12 (1,000 ng/ml) were all added at the beginning of the culture. To examine the effects of these inhibitors on the mRNA expression of osteoclast-related genes, CD14+ cells were cultured in the presence of M-CSF (50 ng/ml) and RANKL (100 ng/ml) for 7 days, and then stimulated with S100A12 (1,000 ng/ml) with or without VIPER (30 μM) and/or the anti-human RAGE antibody (10 μg/ml).

### Tartrate-resistant acid phosphatase (TRAP) staining

TRAP staining was carried out using a kit (Primary Cell, Hokkaido, Japan) according to the manufacturer’s instructions. TRAP-positive multinucleated cells containing more than 3 nuclei were identified as osteoclasts and counted under a microscope (Keyence, Osaka, Japan).

### Bone resorption assay

CD14^+^ monocytes (2×10^5^/well) were cultured on a bone resorption assay plate 48 (PG Research, Tokyo, Japan) in supplemented α-MEM with M-CSF (50 ng/ml), alone or in combination with RANKL (100 ng/ml) and with or without S100A12 (1,000 ng/ml). On day 16, the cells were removed by 5% NaOCl, and then each well was photographed by a microscope camera (Keyence), and the areas of the resorption pits were measured with digital image analysis software (ImageJ 1.49 version from National Institutes of Health, USA).

### Quantitative real-time polymerase chain reaction (qRT-PCR)

Total RNA was isolated using an RNeasy Mini kit (Qiagen, Valencia, CA), and 1 μg of the total RNA was reverse-transcribed with a QuantiTect reverse transcription kit (Qiagen). Quantitative real-time PCR was performed using a QuantiTect SYBR Green PCR kit (Qiagen) with a PikoReal 96 Real-Time PCR system (Thermo Fisher Scientific, Waltham, MA, USA) according to the manufacturer’s protocol. All primer pairs were purchased from Qiagen. Messenger RNA (mRNA) levels were normalized to Glyceraldehyde-3-phosphate dehydrogenase (GAPDH).

### Statistical analysis

Results are expressed as the mean and SD. Statistical analyses were performed using GraphPad Prism 5 (GraphPad Prism Software). Student’s 2-tailed *t*-test was used to evaluate the TRAP-positive cell counts, bone resorption area measurements, and mRNA expression levels of mature osteoclast markers. To evaluate the effects of inhibiting TLR4 and RAGE, one-way analysis of variance and Turkey’s multiple comparison test were used. *P* values less than 0.05 were considered to be statistically significant.

## Results

### S100A12 facilitates osteoclast formation from human CD14^+^ monocytes

**Fig 1:**
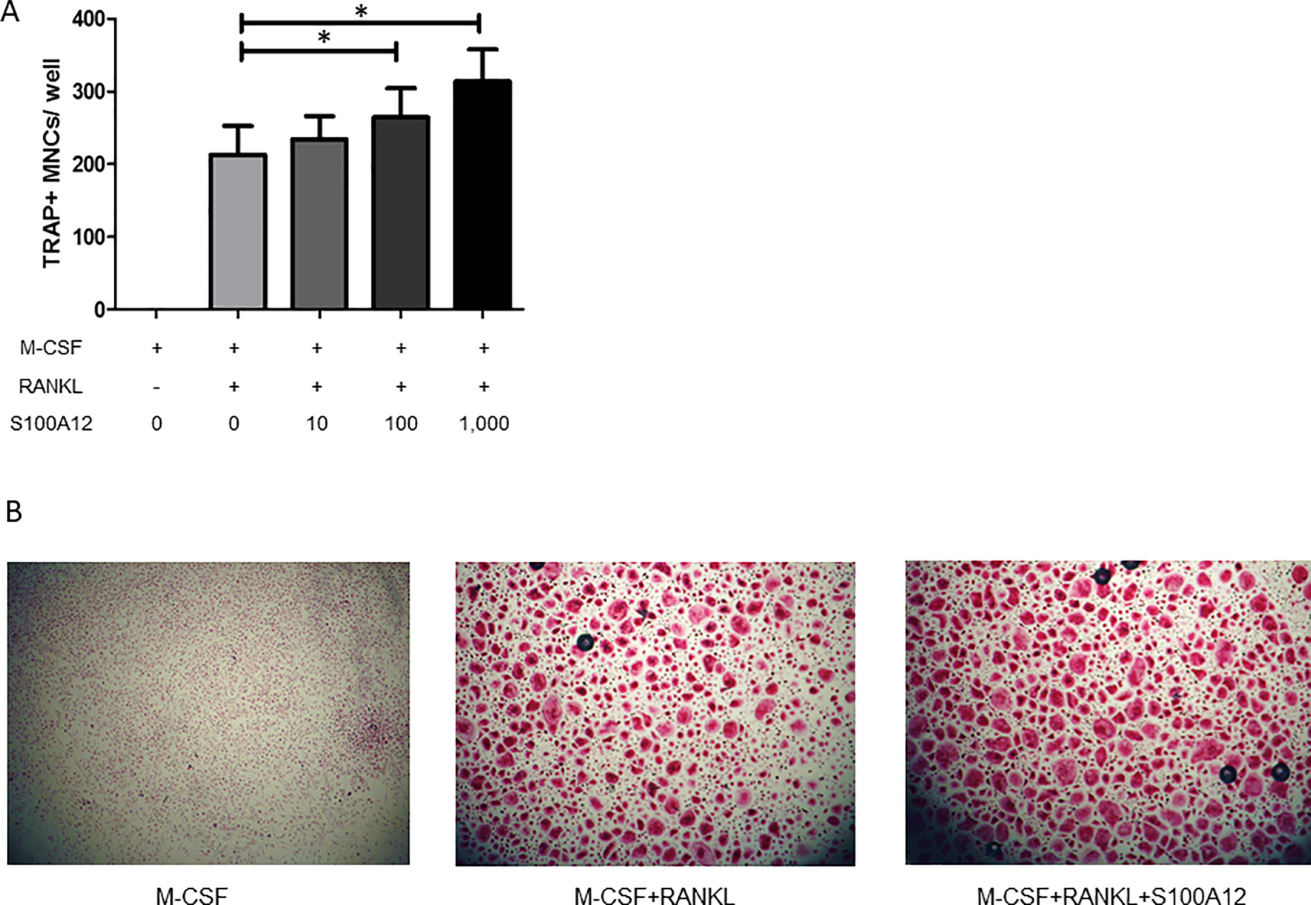
S100A12 stimulates human osteoclast differentiation in a dose-dependent manner. Human blood CD14^+^ monocytes were cultured with M-CSF (50 ng/ml), alone or in combination with RANKL (100 ng/ml) and the indicated concentration of S100A12. Cells were fixed and stained for TRAP after 6 days of culture. **(A)** TRAP-positive multinucleated cells (MNCs) were counted. Values are the mean and SD of the results from 6 independent experiments. * = *p* < 0.05 **(B)** Representative images of TRAP staining (original magnification × 40).

Human blood CD14^+^ monocytes were cultured in the presence of M-CSF (50 ng/ml) and RANKL (100 ng/ml) to promote their differentiation into osteoclasts, and various concentrations of S100A12 were added to examine whether S100A12 enhances osteoclast differentiation. Fig 1B shows representative findings of TRAP staining for the 3 treatment groups. While there was no osteoclast differentiation under M-CSF alone, human blood CD14^+^ monocytes differentiated into osteoclasts in the presence of M-CSF and RANKL. Adding S100A12 facilitated the human osteoclast differentiation in a dose-dependent manner (*p*< 0.05), as shown in Fig 1A. On the other hand, adding S100A12 to human CD14^+^ monocytes in the presence of only M-CSF did not induce osteoclast differentiation (data not shown). Thus, S100A12 enhances RANKL-induced osteoclastogenesis.

### Bone resorption is also stimulated by S100A12

**Fig 2:**
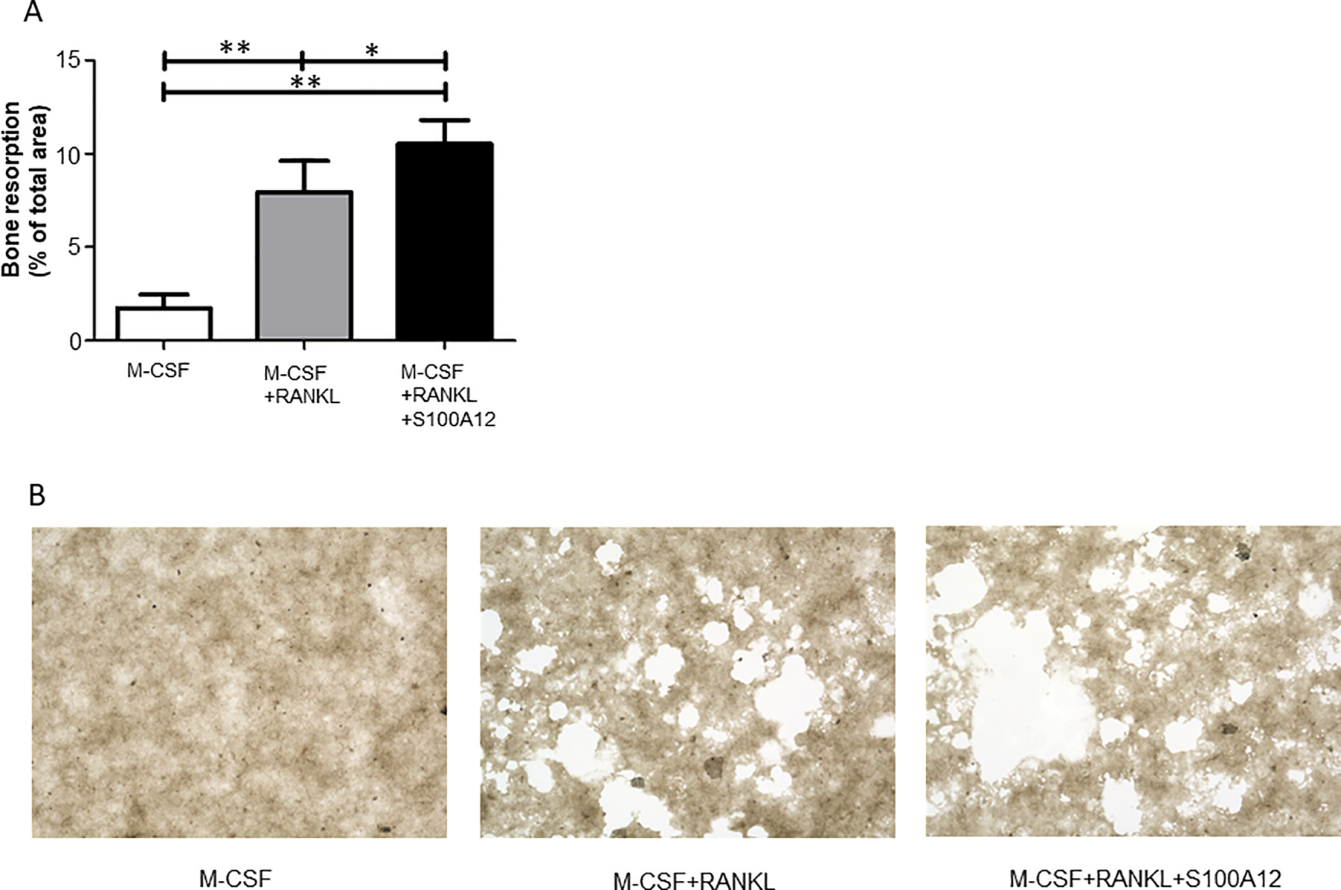
Osteoclasts generated with S100A12 exhibit increased bone resorption. Osteoclasts were generated on a bone resorption assay plate with or without S100A12 (1,000 ng/ml) for 16 days. The resorption lacunae were examined by microscopy. **(A)** The percentage of the resorbed area (i.e., bright areas in **(B)**) was quantified and expressed as the mean and SD of 3 independent experiments. * = *p* < 0.05; ** = *p* < 0.01. **(B)** Representative images of resorption pit formation (original magnification × 200).

We next performed a bone resorption assay to determine whether S100A12 treatment enhances the bone resorption capacity of differentiated osteoclasts. Osteoclasts were generated on a bone resorption assay plate with or without S100A12 (1,000 ng/ml) for 16 days. The added S100A12 appeared to increase the bone resorption levels. Representative patterns of the resorption pit formation are shown in Fig 2B. Image quantification demonstrated that the percentage of bone resorbed by S100A12-stimulated osteoclasts increased compared with those treated with RANKL alone. (Fig 2A).

### S100A12 enhances the expression of markers of mature osteoclasts

**Fig 3:**
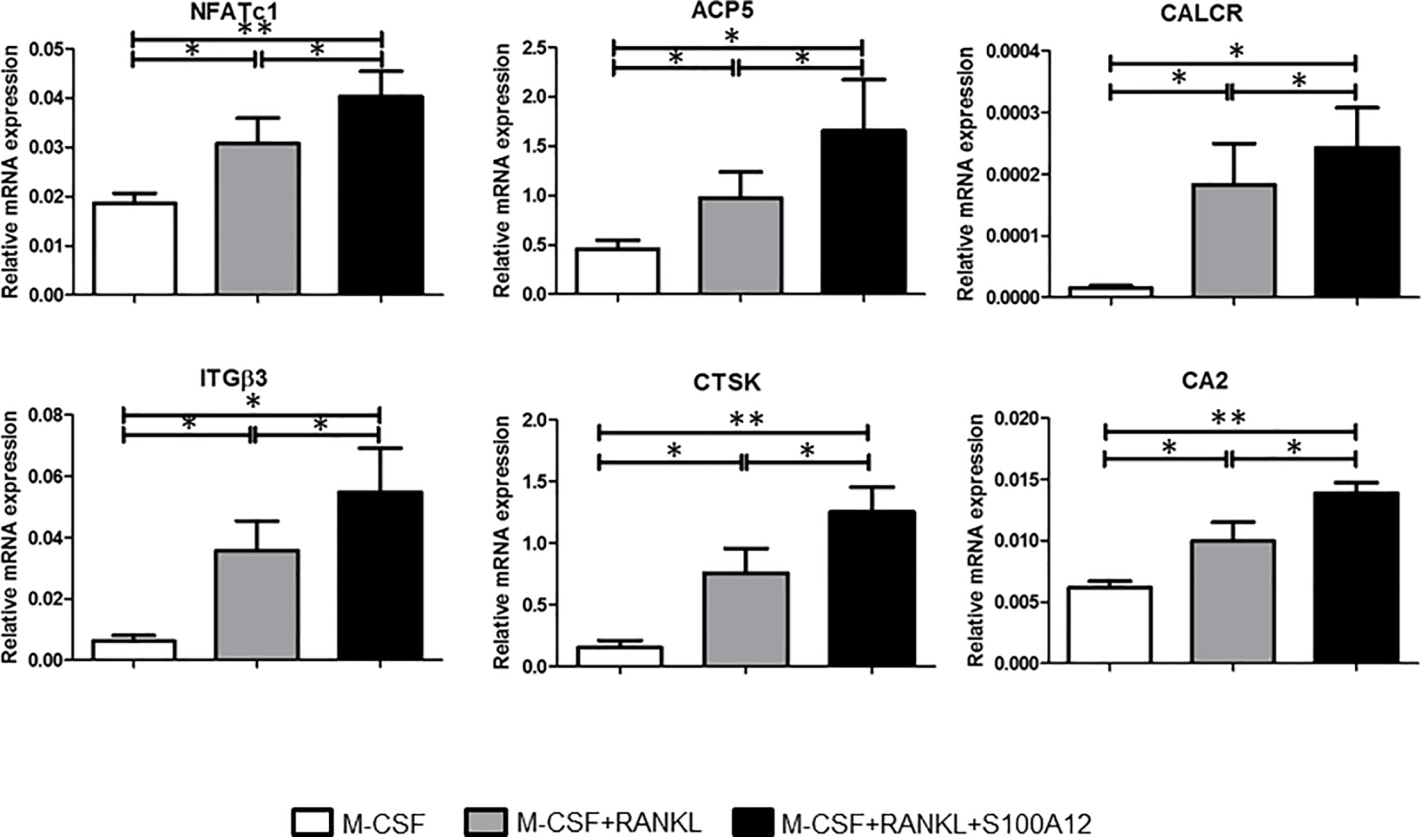
S100A12 stimulates the expression of mature-osteoclast markers. Human CD14^+^ monocytes were cultured in the presence of M-CSF (50 ng/ml), alone or in combination with RANKL (100 ng/ml) and with or without S100A12 (1,000 ng/ml) for 7–9 days. Levels of mRNA expression for nuclear factor of activated T cells c1 (NF-ATc1), TRAP (ACP5), calcitonin receptor (CALCR), integrin β3 (ITGβ3), cathepsin K (CTSK), and carbonic anhydrase II (CA2) were determined by qRT-PCR. Messenger RNA levels were normalized to GAPDH. Values are the mean and SD of 7 independent experiments. * = *p* < 0.05; ** = *p* < 0.01.

Next, the messenger RNA (mRNA) expression levels of several osteoclast-related markers were determined by qRT-PCR. As shown in Fig 3, the expression level of nuclear factor of activated T cells c1 (NFATc1), which is known to act as a master transcription regulator of osteoclast differentiation, was increased by S100A12. In addition, the expressions of mature-osteoclast markers TRAP (ACP5), calcitonin receptor (CALCR), and integrin β3 (ITGβ3) were markedly increased by S100A12 administration (Fig 3A). S100A12 treatment also enhanced the expression levels of cathepsin K (CTSK) and carbonic anhydrase II (CA2), which are known to be critical factors for bone resorption. These findings confirmed the effects S100A12 on osteoclastogenesis at the molecular level.

### TLR4 and RAGE are involved in the bone resorption capacity induced by S100A12

**Fig 4:**
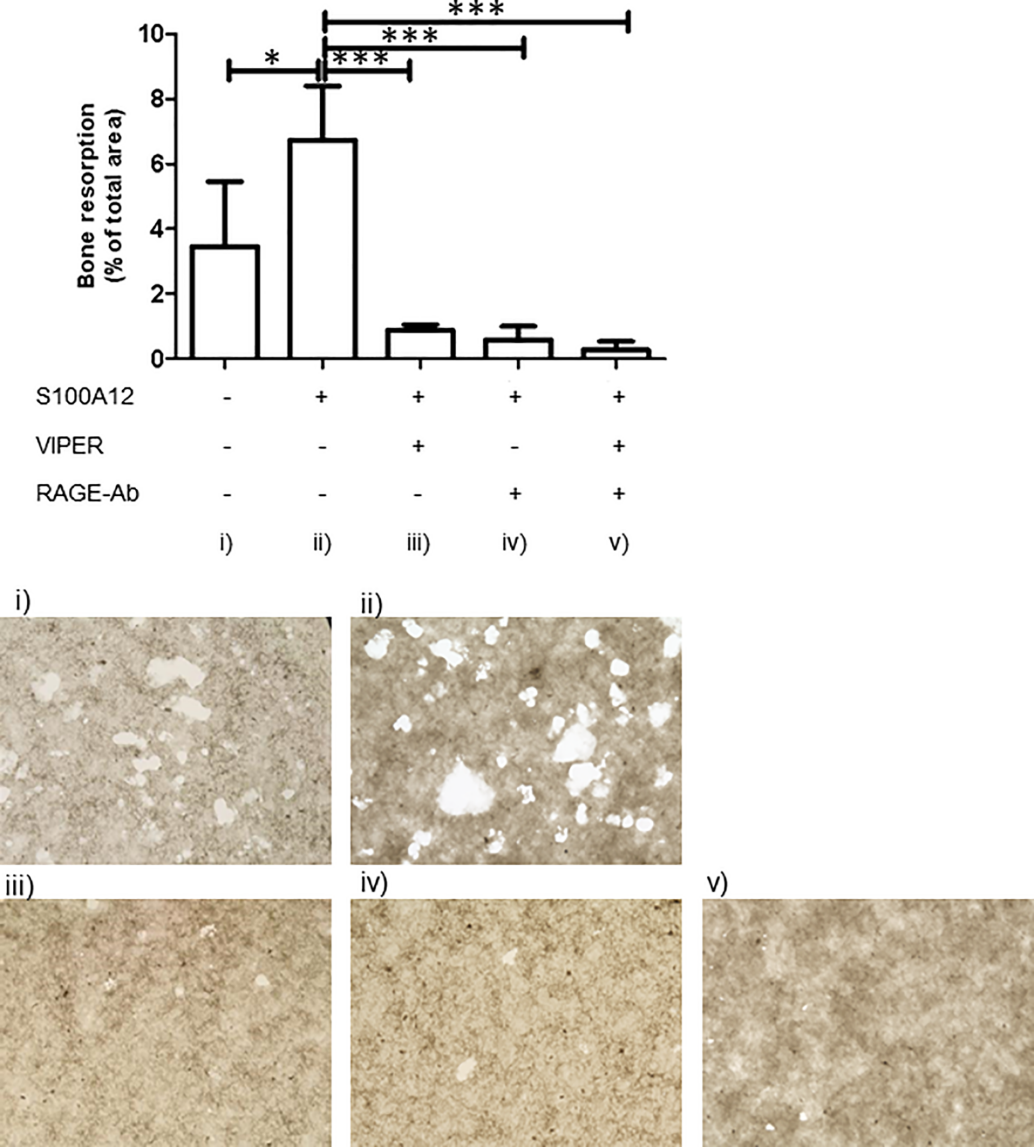
Blocking TLR4 and RAGE reduces the bone resorption capacity of S100A12-induced osteoclasts. Osteoclasts were generated on a bone resorption assay plate with or without S100A12 (1,000 ng/ml), and the additional administration of VIPER (30 μM) and/or an anti-RAGE antibody (10 μg/ml). The resorption lacunae were examined by microscopy after 16 days of culture. **(A)** The percentage of the resorbed area was quantified and expressed as the mean and SD of 3 independent experiments. * = p < 0.05; *** = p < 0.001. **(B)** Representative images of resorption pit formation (original magnification × 200).

S100A12 has been shown to bind multiple receptors, including TLR4 and RAGE. We next examined whether TLR4 or RAGE was critical for the S100A12-induced osteoclast differentiation and bone-resorption capacity. To test this, we performed a bone-resorption assay with S100A12 in the presence of the TLR4 inhibitor VIPER and/or a specific RAGE-blocking antibody. Osteoclasts were induced for 16 days, and VIPER and/or the anti-RAGE antibody was added at the beginning of the culture. As shown in Fig 4, inhibiting RAGE and/or TLR4 markedly suppressed the S100A12-enhanced bone resorption capacity (p< 0.001), indicating that both TLR4 and RAGE are involved in the of S100A12-induced osteoclast function. In these experiments, the S100A12-enhanced osteoclast differentiation was also suppressed by these inhibitors (data not shown).

### S100A12-induced osteoclast functional-marker expression occurs through TLR4 and RAGE

**Fig 5:**
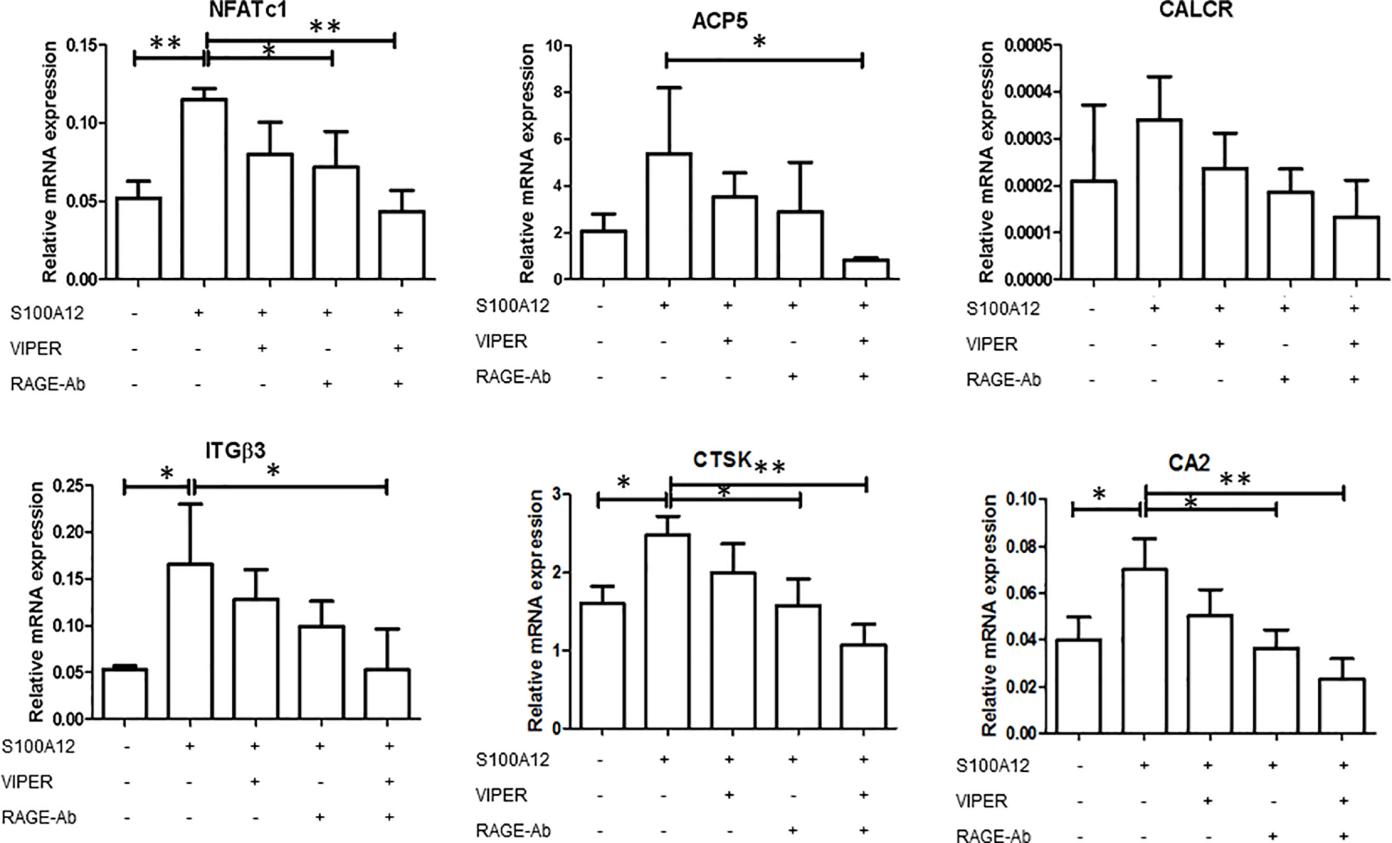
Blocking TLR4 and RAGE inhibits the expression of functional markers in S100A12-stimulated mature osteoclasts. Osteoclast precursors were differentiated for 8 days. On day 7 of differentiation, the cells were stimulated with S100A12 (1,000 ng/ml). VIPER (30 μM) and/or an anti-RAGE antibody (10 μg/ml) were added to the culture 4 hour prior to S100A12 stimulation. After 24 hours of stimulation with S100A12, the mRNA expression levels of NF-ATc1, ACP5, CALCR, ITGβ3, CTSK, and CA2 were examined by qRT-PCR. Messenger RNA levels were normalized to GAPDH. Values are the mean and SD of 3 independent experiments. Statistical analysis was performed using one-way analysis of variance and Turkey’s multiple comparison test. * = *p* < 0.05; ** = *p* < 0.01.

We next examined the roles of TLR4 and RAGE in the S100A12-induced osteoclast-specific mRNA expressions. To this end, human CD14^+^ monocytes were differentiated into osteoclasts with M-CSF and RANKL for 7 days, and then stimulated with S100A12 in the presence of VIPER and/or the specific RAGE-blocking antibody for 24 hours. As shown in Fig 5, blocking RAGE significantly suppressed the S100A12-enhanced NFATc1, CTSK, and CA2 expression (*p*< 0.05, Fig 5). Although VIPER alone did not show significant effects, the combination of VIPER and the anti-RAGE antibody markedly decreased the expression levels of NFATc1, ACP5, ITGβ3, CTSK, and CA2 compared to the no-inhibitors control (*p*< 0.01, Fig 5). Thus, RAGE and TLR play a co-operative role in S100A12’s induction of osteoclast formation.

### The impact of S100A12 on human osteoclast differentiation is similar to that of S100A8 and less than that of S100A9

**Fig 6:**
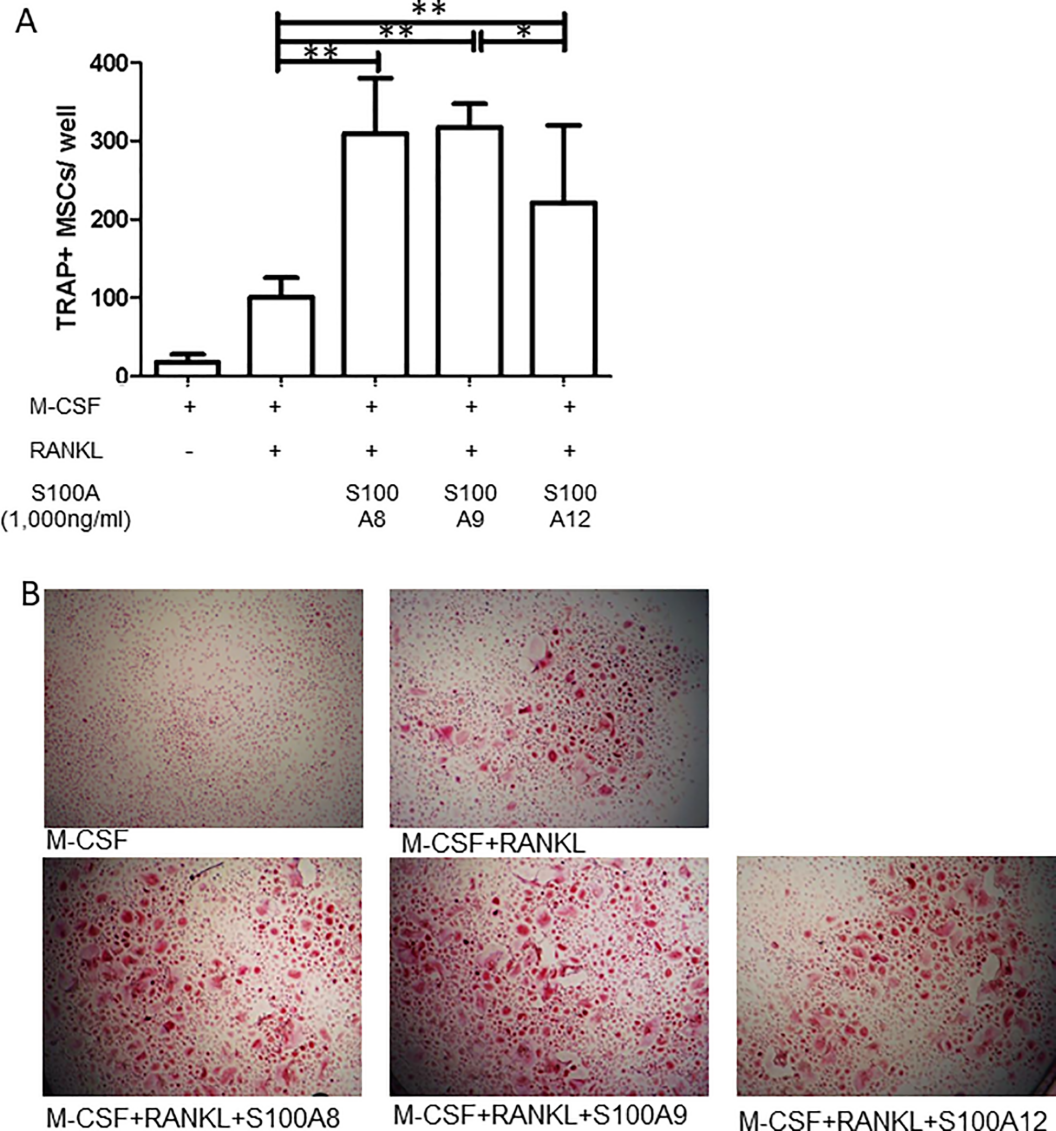
S100A12 has a similar stimulatory effect on human osteoclast differentiation as S100A8, and a weaker effect than S100A9. Human blood CD14^+^ monocytes were cultured with M-CSF (50 ng/ml), alone or in combination with RANKL (100 ng/ml) and S100A8 (1,000 ng/ml), S100A9 (1,000 ng/ml), or S100A12 (1,000 ng/ml). After 6 days of culture, the cells were fixed and stained for TRAP. **(A)** Representative images of TRAP staining (original magnification × 40). **(B)** TRAP-positive MNCs were counted. Values are the mean and SD of the results of 3 independent experiments. * = *p* < 0.05; ** = *p* < 0.01.

Finally, the effects of S100A12 on the differentiation and function of osteoclasts were compared with those of S100A8 and S100A9, which have been shown to induce osteoclast differentiation [13]. We found that the effect of S100A12 was similar to that of S100A8 and slightly less than that of S100A9 (Fig 6). Thus, S100A12 exerts a similar influence on human osteoclastogenesis as S100A8 and S100A9.

## Discussion

In this study, we demonstrated for the first time that S100A12 protein directly enhances osteoclast formation and bone resorption through RAGE and TLR4 in the osteoclast differentiation from human blood CD14^+^ monocytes. Consistent with these findings, S100A12 enhanced the expression of mature osteoclast-related molecules, including NFATc1, ACP5, CALCR, and ITGβ3, and up-regulated CTSK and CA2, both of which are well known to be critical factors in bone resorption [14]. These results show a novel role of S100A12 in inflammatory bone loss.

Inflammatory osteolysis has been ascribed to inflammatory cytokines such as TNF-α, IL-1, and IL-6. These cytokines up-regulate the RANKL expression by osteoblasts and thus facilitate osteoclastogenesis [2]. However, the direct effects of these cytokines on osteoclast differentiation are not simple. The IL-6/sIL-6R system induces osteoclast differentiation in the presence of low-dose RANKL, but inhibits it in the presence of a high (optimal) dose of RANKL [15]. In another report, which examined mouse bone marrow-derived osteoclast formation in vitro, IL-1β enhanced and IL-6 decreased osteoclast formation, and IL-23 and TNF-α showed variable effects depending on the time of administration [16]. Thus, the effects of pro-inflammatory cytokines on osteoclast differentiation are still unclear. However, our report clearly demonstrated that S100A12, along with S100A8 and S100A9, directly enhances osteoclast differentiation in the presence of RANKL, indicating that alarmins are important players in inflammatory osteolysis.

Alarmins use various receptors. HMGB1 regulates RANKL-induced osteoclastogenesis through RAGE [17], while S100A8 enhances osteoclastic bone resorption through TLR4 [13]. Our results indicated that both RAGE and TLR4 are critically involved in S100A12’s effects on osteoclast differentiation and bone resorption capacity (Fig 4). In the short-term S100A12 stimulation experiment in Fig 5, both RAGE and TLR4 were critical for suppressing osteoclast-related genes, with RAGE having a dominant effect on the expression of NFATc1, CTSK, and CA2. The S100A12-RAGE interaction is reported to trigger cytokines and cellular activation [17]. Our results indicated that this interaction also triggers inflammatory bone resorption.

Elevated levels of other S100 proteins, including S100A8 and S100A9, are observed in RA serum and synovial fluid [9]. In addition, an anti-RAGE antibody decreased the osteoclastogenic potential in osteoarthritic fibroblast-like synovial cells, indicating that S100A-RAGE is involved in the inflammation and osteoclastogenesis in arthritis [18]. Here we demonstrated that S100A12 induced osteoclast differentiation almost to the same extent as S100A8 and S100A9. These findings suggest that inhibiting S100A12, S100A8, and S100A9, could be an effective therapeutic strategy for osteoclast-related disease, such as the erosive form of RA and the systemic bone loss in various inflammatory conditions. On the other hand, S100A12 is associated with vascular calcification through an osteogenic gene regulatory program [19]. Thus, S100A12 plays a bidirectional role in bone formation and in the resorption of chronic inflammation by inducing osteoblastic changes and osteoclast differentiation.

In conclusion, we demonstrated that S100A12 is involved in RANKL-induced osteoclast differentiation and bone resorption. S100A12 and RAGE represent potential therapeutic targets for inflammatory bone resorption.
